# Editorial: A lifespan approach towards paediatric pain management: from neonates to young adults

**DOI:** 10.3389/fpain.2026.1933896

**Published:** 2026-08-18

**Authors:** L. Caes, J. McParland, H. Durand

**Affiliations:** 1 Division of Psychology, Faculty of Natural Sciences, University of Stirling, Stirling, United Kingdom; 2 Department of Psychology, School of Health and Life Sciences, Glasgow Caledonian University, Glasgow, United Kingdom

**Keywords:** continuity of care, lifespan, paediatric pain, personalisation, transition

Given the interdisciplinary nature of paediatric pain management, especially in the case of chronic pain, continuity and personalisation of care is key to ensure that families can engage with and navigate such complex healthcare systems, involving multiple stakeholders and various transitions across the lifespan, as smoothly as possible (1). The World Health Organisation (WHO) defines continuity of care as “*the degree to which a series of discrete health care events is experienced by people as coherent and interconnected over time and consistent with their health needs and preferences*” (2). Personalised, continuity of care has many benefits such as reducing healthcare costs, hospital admissions, emergency care visits, and increased satisfaction with care (2, 3). A recent scoping review on continuity of care within paediatric chronic illnesses (including chronic pain conditions) (1) developed a process model of high-quality care coordination in paediatric patients, characterisizing high-quality care coordination by (1) setting clear responsibilities, (2) effective and reliable communication between professionals as well as with caregivers, (3) formalized, written care coordination plans, (4) flexible and child-centred care through building trust and relationships, and (5) involvement of community-based structures (e.g., schools). However, a key issue with continuity of care is that it is often threatened or broken when a young person transitions into adult care (4). While a consensus on successful transition outcomes is lacking, optimal transition should not only support better engagement and satisfaction with pain management services but also improve well-being and attainment of relevant vocational and social milestones (4). There is an urgent need to better understand this transition process and ensure that the continuity and personalisation of care stretches across paediatric and adult healthcare settings, which a lifespan approach would bring.

The aim of this research topic “*A Lifespan Approach towards Paediatric Pain Management: from Neonates to Young Adults*” was to highlight the need for the paediatric pain community to adopt a wider lifespan approach towards the paediatric pain experience to support the continuity of care from childhood into adulthood. A lifespan approach should feature a strong collaboration between paediatric and adult pain services as well as community-based services to support continuity of care and coordination in managing acute and chronic pain experiences through a young person's developmental trajectory. The research topic was inspired by the same theme that was featured at the International Symposium on Paediatric Pain held in Glasgow in June 2025, with the editors as the co-chairs of the local organising committee of ISPP2025. While several articles published within this research topic represent presentations and discussion that occurred at ISPP2025, the content is not limited to the ISPP2025 coverage.

In an effort to showcase the relevance of lifespan-focused continuity and personalisation of care, the research topic contains 10 publications illustrating the variety of innovative work being conducted around paediatric pain from neonates to young adulthood across a wide range of pain symptoms, e.g., cancer pain (Caralho et al.), chronic post-surgical pain (Ceniza-Bordallo et al.; Curry et al.), pain due to medical procedures (Lugli et al.), migraines (Caes et al.), dysmenorrhoea (Wang and Guo), and general chronic pain (Niburski et al.; Nishat et al.; Swidrovich et al.). The studies reflect a wide range of methodologies to tackle and explore the consistency, continuity and collaboration in care, ranging from panel transcripts (Swidrovich et al.), opinion pieces (Wang and Guo) and systematic reviews (Ahmad et al.) to qualitative explorations of lived experiences (Caes et al.), real-life clinical evaluations [such as quality improvement (Niburski et al.) and pharmacoepidemiologic evaluations (Caralho et al.)] and cross-sectional multi-method analyses (Ceniza-Bordallo et al.; Curry et al.; Lugli et al.; Nishat et al.). Regardless of the diversity in methods, age and pain focus, all publications explored the wider sociological impact of or influences on pain, such as adverse events, gender identity, and transition into adulthood, with the common goal of improving our assessment and/or management of paediatric pain at any stage of development to prevent further lifelong challenges due to either acute and chronic childhood pain experiences. Key recommendations related to the lifespan and continuity of care approach across the articles are as follows:

- Theoretical frameworks, such as the framework proposed by Wang and Guo for dysmenorrhea prevention and control, could underpin clinical approaches that prevent a lifetime challenge of dealing with chronic pain. Importantly, these frameworks need to be informed by the lived experiences of young people across all key stages in development (including young adulthood, Caes et al.) and all gender identities, thereby ensuring appropriate reflection of intersectionality and challenging systemic norms (Swidrovich et al.).
- A clear understanding of different pathways of chronic pain experiences (such as post surgical chronic pain pathways, Ceniza-Bordallo et al.), which acknowledge wider psychosocial factors [such as parental upheaval (Curry et al.) and mental health (Nishat et al.)], are crucial to support the development of personalised, lifespan approaches to paediatric chronic pain management.
- Appropriate pain management strategies need to start in neonates, with effective pain assessment and interdisciplinary pain management (i.e., including pharmacological and care-model approaches) this early on having the potential to significantly reduce the strain on healthcare, by not only reducing lifelong impairment but also healthcare reliance and usage across the lifespan (Ahmad et al., Lugli et al.)
- A clear understanding of the effectiveness as well as adverse events associated with pharmacological approaches of pain is key to support personalised and transparted care plans that can support a strong continuity of care by supporting patient-involved and -centred decision making processes (Caralho et al.; Niburski et al.).

In sum, we believe this research topic highlights important endevours towards a lifespan and personalised approach to support continuity of care, while identifying important next steps and gaps in the literature. A key gap that remains for future research is the availability of longitudinal, prospective approaches exploring pain experiences, impact and management across the entire developmental trajectory, in diverse samples in terms of pain characteristics, sociodemographic background and gender identity.
